# DDX3X Syndrome: Clinical, Neuroimaging, AI-Assisted Facial Profiling and Genotype–Phenotype Correlations

**DOI:** 10.3390/genes17050551

**Published:** 2026-05-05

**Authors:** Sara Hadj Sadok, Alicia Irene Serra, Leticia Diana Pias-Peleteiro, Diana Salinas Chaparro, Clara Xiol, Judith Armstrong, Encarna Guillén-Navarro, Antonio F. Martínez-Monseny

**Affiliations:** 1 Department of Medical Genetics, Hospital Sant Joan de Déu, 08950 Esplugues de Llobregat, Spain; sara.hadj@sjd.es (S.H.S.);; 2 Center for Genomic Medicine, Parc Taulí Hospital Universitari, Institut d’Investigació i Innovació Parc Taulí (I3PT-CERCA), Universitat Autònoma de Barcelona, 08193 Sabadell, Spain; 3 Institut de Recerca Sant Joan de Déu, 08950 Esplugues de Llobregat, Spain; 4 Centro de Investigación Biomédica en Red de Enfermedades Raras, 28029 Madrid, Spain; 5 Departament of Medical Genetics, Hospital Clínico Universitario Virgen de la Arrixaca, 30120 El Palmar Murcia, Spain

**Keywords:** DDX3X syndrome, neurodevelopmental disorder, Face2Gene, artificial intelligence, facial recognition

## Abstract

**Background/Objectives**: DDX3X syndrome (MIM#300958) is a neurodevelopmental disorder associated with intellectual disability, language impairment, and a characteristic neurobehavioral phenotype that predominantly affects females. Although dysmorphic features have been reported, a consistent facial phenotype and clear genotype–phenotype correlations have not been established. **Methods**: We conducted an observational, ambispective, descriptive study including patients aged 0–18 years with a molecular diagnosis of DDX3X. Clinical, standardized facial images, neurobehavioral, neuroimaging, and molecular data were collected. Automated facial analysis was performed using Face2Gene after algorithm training. **Results**: Of 11 identified patients, 9 were included (8 females); 8 variants were *de novo* and 4 novel. Two variants of uncertain significance underwent in silico analysis. Frequent facial features included thin upper lip (9/9), tapered chin (8/9), long uniform eyebrows (8/9), short neck (8/9), and long face (6/9). After training, Face2Gene identified DDX3X syndrome in 92% of cases within the top 5 suggestions, supporting its utility as a diagnostic aid. All females had intellectual disability and language disorder; 66% presented sleep disturbances and aggressive behavior. Neuroimaging revealed ventricular dilatation (5/9) and corpus callosum hypoplasia (3/9). Loss-of-function variants were associated with greater clinical severity. **Conclusions**: This series suggests a recognizable facial phenotype of DDX3X syndrome and supports a possible genotype–phenotype correlation. Further studies are needed to confirm these findings.

## 1. Introduction

DDX3X syndrome (MIM#300958) is a neurodevelopmental disorder caused by pathogenic variants, described by Snijders Blok et al. [1] as one of the most frequent monogenic causes of intellectual disability (ID) in females, accounting for 1–3% of unexplained cases in their series [1]. With currently more than 1000 females diagnosed worldwide, it represents one of the most common X-linked intellectual disability syndromes [2]. The gene maps to chromosome Xp11.4 and encodes a 662-amino acid ATP-dependent RNA helicase organized into two conserved domains: the helicase ATP-binding domain (residues 211–403), which harbors the DEAD-box motif (residues 247–350), and the helicase C-terminal domain (residues 414–575) [3,4] DDX3X participates in virtually all steps of RNA metabolism, from transcriptional regulation and pre-mRNA processing to translation initiation and the assembly of cytoplasmic stress granules [5].

The mechanism underlying the phenotypic variability in DDX3X syndrome remains incompletely understood. Truncating variants are generally attributed to haploinsufficiency [1], while functional studies have associated a subset of missense variants with more severe neurological outcomes, including polymicrogyria and profound intellectual disability, through a proposed dominant-negative disruption of helicase activity [6,7]. This apparent paradox makes genotype–phenotype prediction challenging and has not been fully resolved.

Clinical manifestations are characterized by intellectual disability of variable severity (mild to severe), hypotonia, language delay with absent speech in up to 51% of girls at age 5 years, sleep disturbances, and behavioral features including autism spectrum traits, attention deficit, impulsivity, and self- or hetero-aggressive behaviors [1,6,8]. Although dysmorphic facial features have been reported across multiple cohorts, there is no consistent facial gestalt associated with the syndrome [2,9].

Although DDX3X syndrome predominantly affects females, male cases have been increasingly reported. A recent study presenting the largest male cohort to date confirmed that most affected males carry missense variants and that no truncating variants have been identified in males, consistent with the presumed embryonic lethality of complete *DDX3X* loss-of-function in the hemizygous state [2]. The clinical phenotype in males overlaps with that of females but is generally less severe, and carrier mothers are often asymptomatic or mildly affected [2,10,11].

Despite the growing number of clinical descriptions, important gaps remain in the literature, particularly regarding potential genotype–phenotype correlations. In the present study, we describe a case series of nine patients with DDX3X-related neurodevelopmental disorder, integrating genetic and phenotypic data.

## 2. Methods

We conducted an observational, ambispective, and descriptive study. Patients of both sexes aged 0–18 years with a molecular diagnosis of DDX3X syndrome, evaluated at the Department of Medical Genetics of Hospital Sant Joan de Déu (Barcelona, Spain).

Clinical, neurobehavioral, neuroimaging, and molecular data were collected from electronic medical records and direct clinical evaluation. All patients underwent in-person assessment by clinical genetics and neurogenetics specialists. Standardized frontal facial photographs were obtained with written informed consent. Automated facial analysis was performed using Face2Gene RESEARCH platform.

Molecular diagnosis was established by next-generation sequencing (whole-exome or clinical exome sequencing) in all patients. Variants were classified according to ACMG/AMP guidelines.

The study was conducted in accordance with the Declaration of Helsinki (Fortaleza revision, 2013) and approved by the Ethics and Research Committee of Hospital Sant Joan de Déu, Barcelona. Written informed consent was obtained from the legal guardians of all participants prior to inclusion. Consent for the publication of facial photographs was obtained separately.

## 3. Results

### 3.1. Patient Cohort Overview

Of 11 patients diagnosed with DDX3X syndrome at our department during the study period, nine were included in the final analysis (eight female, one male; 82%/18%). Two patients were excluded: one due to difficulties reaching the family and unwillingness to participate in the study. The age range at evaluation was 3 years 10 months to 15 years 6 months, with a mean of 8 years 8 months. Eight of nine variants were *de novo* and one variant, identified in the male patient (Patient 9), was maternally inherited from an asymptomatic carrier mother.

**Figure 1:**
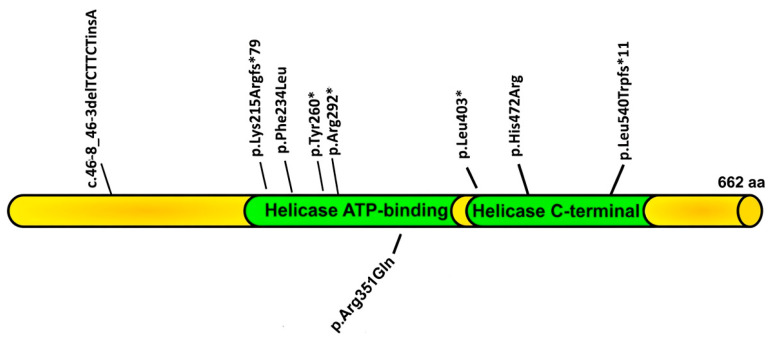
*DDX3X* variants identified in this series.

Nine variants were identified (Table 1; Figure 1). Eight are located in coding exons and one is intronic. By protein domain: six variants reside in the helicase ATP-binding domain (Patients 2, 3, 4, 5, 8, 9), two in the helicase C-terminal domain (Patients 1 and 6), and one affects a splice acceptor site intronic to the helicase ATP-binding domain (Patient 7).

Regarding predicted molecular consequence, four variants are predicted to result in a premature stop codon (44.4% combined): three through direct nonsense substitution and one through a frameshift introducing a stop codon 11 residues downstream. The remaining variants comprised three missense substitutions (33.3%), one frameshift (11.1%), and one intronic *indel* at a splice acceptor site (11.1%). Four of the nine variants have not been previously reported and are therefore novel.

By ACMG/AMP classification: four variants are Pathogenic (44.4%), three are Likely Pathogenic (33.3%), and two are Variants of Uncertain Significance (VUS; 22.2%). Eight of the nine variants are *de novo* (88.9%). The male patient (P9) carries a maternally inherited missense variant (p.Arg351Gln).

In silico analysis of the two variants of uncertain significance was performed.

### 3.2. In Silico Analysis of VUS

Patient 2—c.700T>C; p.Phe234Leu (helicase ATP-binding domain).

This variant is absent from GnomAD and CSVS population databases and has no prior entry in ClinVar or HGMD. Multiple in silico pathogenicity tools were applied: PolyPhen-2 predicted probably damaging; CADD score 27.1; seven of ten MobiDetails predictors (including SIFT, REVEL, and ClinPred) classified the variant as deleterious. Protein structural analysis with DynaMut2 and MIZTLI predicted destabilization. Conservation analysis via MetaDome indicated the affected residue is intolerant to variation. One predictor (Missense3D) did not predict gross structural disruption. Based on these findings and the *de novo* occurrence in a patient with a compatible phenotype, the variant was reclassified as likely pathogenic.

Patient 7—c.46-8_46-3delTCTTCTinsA (intronic splice acceptor, pre-exon 1).

This variant is absent from all population and clinical databases. Splice prediction was performed with MaxEntScan, SPiP, and NetGene2. SPiP predicted 100% disruption of the native splice acceptor site. MaxEntScan showed a score variation of −245% between wild-type and mutant sequences, with a raw score difference of −14. NetGene2 confirmed loss of the native acceptor site in the mutant sequence; the nearest alternative acceptor site was identified 1719 bp downstream with substantially reduced confidence (score 0.15 vs. 0.64 wild-type), predicting potential skipping of exons 2 and 3 of the DDX3X transcript. The ACMG classification remains VUS pending RNA-level functional confirmation due to phenotypic compatibility.

### 3.3. Facial Phenotype and AI-Assisted Diagnosis

Systematic dysmorphological examination identified a consistent set of facial features across the majority of patients (Table 2). The most prevalent findings were: thin upper lip (9/9; 100%); low-set thumb insertion (9/9; 100%); flat philtrum (8/9; 89%); tapered chin (8/9; 89%); short neck (8/9; 89%); uniform long eyebrows (8/9; 89%); broad nasal bridge (7/9; 78%); long face(6/9; 67%); broad forehead (6/9; 67%); upslanting palpebral fissures (6/9; 67%); bulbous nasal tip (6/9; 67%); and narrow nares (6/9; 67%). Deep-set eyes and long philtrum were each present in 5/9 patients (56%). Overweight (BMI ≥ 85th percentile for age) was observed in 5/9 (56%) and microcephaly in 3/9 (33%).

While the overall phenotype of our patient overlaps with previously reported cases, uniform long eyebrows, proximal insertion of the thumb, and short neck have not been previously described in the literature.

To assess whether facial recognition was possible, photographs of patients were uploaded to the Face2Gene platform. Prior to Face2Gene training, DDX3X syndrome appeared within the top-5 differential diagnoses. Following algorithm training with 21 additional photographs provided by families affiliated with Asociación DDX3X España, the recognition rate increased to 92%. The most frequent co-occurring differentials included Rett syndrome, Noonan syndrome, Turner syndrome, 22q11.2 deletion syndrome, and Smith-Magenis syndrome, reflecting phenotypic overlap between DDX3X syndrome and other neurodevelopmental conditions. The male patient was not recognized by the algorithm in either phase.

These findings suggest that DDX3X syndrome may have a recognizable facial phenotype.

**Figure 2:**
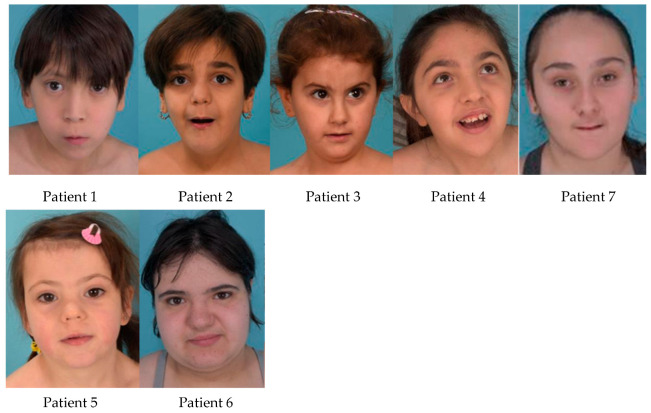
Frontal facial photographs of the nine patients included in this series. Upper row (left to right): Patients 1, 2, 3, 4, and 7. Lower row (left to right): Patients 5 and 6.

The facial photographs are shown in Figure 2.

### 3.4. Neurodevelopmental and Cognitive-Behavioral Phenotype

Global developmental delay was present in all eight female patients (8/8; 100%), with predominantly expressive language impairment ranging from non-verbal to short incomplete sentences. All affected females produced their first words after 3 years of age. Among those who developed verbal communication, two-word phrases emerged after 7 years of age. Three of nine patients overall had intelligible speech at evaluation (3/9; 33%). The male patient (P9) had age-appropriate language and a neuropsychological profile within the low-average cognitive functioning (IQ 82).

Autonomous gait was achieved by all patients, ranging from the age of 15 months in the male patient to 4 years and 6 months in the most severely affected females. Hypotonia was present in all female patients (8/8; 100%) and absent in the male. Gait abnormalities were noted in 3/9 (33%). Epilepsy was documented in one patient (1/9; 11%), with focal seizures onset at 3 months of age and status epilepticus at 4 months, remaining seizure-free after the age of 17 months and allowing anti-seizure medication to be suspended.

Autism spectrum disorder (ASD) features were reported in all female patients (8/8; 100%). Formal ASD diagnosis confirmed by ADOS-2 assessment was established in 3/9 patients (33%). Sleep disturbances were present in 6/9 (67%), predominantly maintenance insomnia, with a variable degree of severity. One patient (P4) averaged only 5 h of continuous sleep per night. Brief Infant Sleep Questionnaire (BISQ) were administered revealing absence of daytime napping in all patients despite nocturnal sleep fragmentation. Behavioral abnormalities with aggressive traits were present in 5/9 (56%).

Brain MRI was performed on all nine patients. Ventricular enlargement was the most frequent finding (5/9; 56%), followed by corpus callosum hypoplasia (3/9; 33%) and intracranial cysts (2/9; 22%), the latter including a Blake’s pouch cyst and a small periatrial neuroglial cyst. No patients in this series showed polymicrogyria or other malformations of cortical development (Table 3). Additional features previously reported in DDX3X syndrome, including skin abnormalities, recurrent infections, auditory abnormalities, and congenital heart defects [6,7,12], were not systematically assessed in this cohort. Their documentation therefore relied on retrospective medical record review, and their prevalence may be underestimated.

### 3.5. Phenotypic Severity and Variant Type

The most severe combined neurodevelopmental phenotype was observed in Patients 4 and 7. Both presented with profound intellectual disability, aggressive behavior, and severe sleep disturbance, and both shared a notably similar facial profile including broad forehead, flat philtrum, thin upper lip, tapered chin, short neck, and uniform long eyebrows. Patient 4 carries a nonsense variant (p.Tyr260*) within the DEAD-box motif of the helicase ATP-binding domain. Patient 7 carries an intronic indel (c.46-8_46-3delTCTTCTinsA) at the splice acceptor site preceding exon 1, predicted by multiple tools to result in loss of the native splice site. Both variants are predicted to abolish or severely reduce functional DDX3X protein production. At the other end of the spectrum, the male patient (P9) carrying a maternally inherited missense variant presented with low-normal cognition (IQ 82), age-appropriate language, and no hypotonia.

## 4. Discussion

DDX3X syndrome remains underdiagnosed, partly due to phenotypic variability and the absence of a clearly defined facial gestalt. This case series of nine patients evaluated at a single tertiary center addresses two of the key gaps identified in the current literature, offering new data on facial phenotype and the clinical utility of AI-assisted diagnosis.

### 4.1. Novel Variants and the Expanding Molecular Landscape

Four of nine variants identified in this series are novel, adding to a mutational spectrum in which pathogenic alleles are approximately equally distributed between protein-truncating and missense variants. All nine variants map to the two functional helicase domains (the ATP-binding domain and the C-terminal domain), consistent with the established observation that pathogenic *DDX3X* variants cluster within these regions, where they are predicted to directly impair RNA-unwinding activity [6,13].

Patient 9, the sole male in this series, carries a maternally inherited missense variant (p.Arg351Gln) from an asymptomatic carrier mother, and presented with a mild phenotype, characterized by low-normal cognition (IQ 82), age-appropriate language, and no hypotonia. This is consistent with the established model of sex-differential dosage sensitivity, in which hypomorphic variants produce a clinical phenotype in hemizygous males while remaining silent in heterozygous females who retain a functional second allele [2,10,11]. Importantly, none of the truncating *de novo* variants identified in females in this and other series have been reported in hemizygous males, consistent with the hypothesis that complete DDX3X loss-of-function is embryonically lethal in males [2].

### 4.2. In Silico Characterization of VUS: Utility and Limitations

Two variants were classified as VUS at the time of diagnosis and underwent structured in silico analysis. For Patient 2 (p.Phe234Leu), convergent evidence from multiple independent pathogenicity prediction tools, including a CADD score of 27.1, protein structural destabilization predictions, and evolutionary conservation analysis indicating intolerance to variation at the affected residue, supported reclassification to likely Pathogenic. The *de novo* occurrence in a patient with a compatible phenotype provided additional supporting evidence under ACMG criteria PM6 and PP3. However, not all tools were concordant: Missense3D did not predict gross structural damage, illustrating a recognized limitation of computational approaches, since prediction tools are trained on different datasets and may diverge for variants with moderate functional effects. This reclassification should therefore be considered provisional until confirmed by cellular functional assays.

For Patient 7 (c.46-8_46-3delTCTTCTinsA), the predicted severity of splice disruption is consistent with a severe loss-of-function mechanism that would result in skipping of exons 2 and 3 of the DDX3X transcript. SPiP predicts 100% loss of the native acceptor site, MaxEntScan shows a score variation of −245%, and an alternative acceptor site is located 1719 bp downstream. Nevertheless, ACMG classification remains VUS because splice predictions have not been validated at the RNA level. This case illustrates a broader diagnostic gap: intronic variants near splice sites are increasingly detected by genomic sequencing but remain under-characterized, as routine RNA studies are not standard in most clinical genetics workflows. RNA-level analysis, specifically RT-PCR or RNA sequencing on patient-derived cells, is recommended as a priority follow-up for this patient.

### 4.3. Facial Phenotype and AI-Assisted Diagnosis

Our dysmorphological assessment identified several facial and somatic features not previously systematically described in *DDX3X* patients, including uniform long eyebrows, pointed chin, short neck, and proximal placement of thumb. The overall facial gestalt, characterized by thin upper lip, flat philtrum, broad nasal bridge, and upslanting palpebral fissures, was internally consistent across the cohort. While facial phenotypes have been noted since the original description [1], no consistently recognizable gestalt has been established, partly owing to heterogeneity across cohorts and the absence of standardized dysmorphological assessment protocols. Our findings suggest that several features are consistent across independent series even when frequency estimates differ, likely reflecting variation in ascertainment rather than genuine inter-cohort differences.

The Face2Gene training experiment produced a clinically meaningful result: adding 21 photographs to the training dataset improved the recognition rate by 30 percentage points, with DDX3X syndrome entering the top differential for all eight female patients post-training, including those carrying VUS variants. This supports the use of AI-assisted facial analysis as a diagnostic adjunct, particularly in settings where specialist dysmorphology expertise is limited or genetic testing results are pending. The failure to recognize the male patient in either phase is consistent with the reduced or distinct facial phenotype reported in affected males [2], and highlights a known limitation of current AI tools: models trained predominantly on female patients may not generalize to the male phenotype without sex-stratified training data.

The application of AI-assisted facial phenotyping tools in neurodevelopmental disorders is a rapidly growing field. Gurovich et al. demonstrated that the DeepGestalt algorithm underlying Face2Gene achieved 91% top 10 accuracy across more than 200 syndromes, including multiple neurodevelopmental conditions [14]. Our findings support the growing utility of this technology in rare neurodevelopmental disorders, and highlight the potential of collaborative patient registries and family associations in building the training datasets necessary for syndrome-specific recognition.

### 4.4. Variant Type, Predicted Mechanism, and Neurodevelopmental Severity

In this series, the two patients meeting predefined severity criteria both carry predicted loss-of-function variants rather than missense. This is noteworthy in the context of published genotype–phenotype data, in which a subset of missense variants and in-frame deletions have been associated with more severe phenotypic outcomes than protein-truncating variants [6,12,15].

The basis for the clinical severity observed in these two patients cannot be determined from the available data. Notably, Patient 7’s variant remains classified as VUS pending RNA-level confirmation, which limits the strength of any genotype–phenotype inference.

Larger cohorts and functional studies will be needed to confirm these observations.

## 5. Conclusions

This case series describes nine patients with DDX3X-related neurodevelopmental disorder, reporting novel variants and expanding knowledge of a potentially recognizable facial phenotype supported by AI-assisted diagnosis, while associating predicted loss-of-function variants with greater clinical severity.

## Figures and Tables

**Table 1:** *DDX3X* variants identified in the nine patients of this series.

Patient	cDNA (NM_001356.5)	Protein Change	Type	Domain	Inheritance	ACMG Classification
P1	c.1415A>G	p.His472Arg	Missense	Helicase C-terminal	*De novo*	Pathogenic (PP2, PP3, PP5, PM1, PM2, PM5)
P2	c.700T>C	p.Phe234Leu	Missense	Helicase ATP-binding	*De novo*	VUS → reclassified LP * (PM2, PP3-supp)
P3	c.644_645delAA	p.Lys215Argfs*79	Frameshift	Helicase ATP-binding	*De novo*	Likely Pathogenic (PVS1, PM2, PP3)
P4	c.780T>G	p.Tyr260*	Nonsense	DEAD-box motif (ATP-binding)	*De novo*	Pathogenic (PS4, PVS1, PM2)
P5	c.874C>T	p.Arg292*	Nonsense	Helicase ATP-binding	*De novo*	Pathogenic (PS2, PVS1, PM2)
P6	c.1618delC	p.Leu540Trpfs*11	Frameshift	Helicase C-terminal	*De novo*	Likely Pathogenic (PVS1, PM2)
P7	c.46-8_46-3delTCTTCTinsA	p.? (splice acceptor)	Splicing	Splice acceptor, pre-exon 1	*De novo*	VUS (PM2)—Funcional studies pending.
P8	c.1208T>A	p.Leu403*	Nonsense	Helicase ATP-binding	*De novo*	Likely Pathogenic (PVS1, PM2)
P9	c.1052G>A	p.Arg351Gln	Missense	DEAD-box motif (ATP-binding)	Maternal	Pathogenic (PS4, PM1, PP2, PM2, PM5)

* Reclassified from VUS to Likely Pathogenic following in silico analysis. ACMG: American College of Medical Genetics and Genomics; LP: Likely Pathogenic; P: Pathogenic; VUS: Variant of Uncertain Significance. ACMG evidence criteria: PVS1: Pathogenic Very Strong (null variant in gene where loss of function is a known disease mechanism); PS2: Pathogenic Strong (de novo variant in patient with disease and no family history); PS4: Pathogenic Strong (increased prevalence of variant in affected individuals); PM1: Pathogenic Moderate (located in critical functional domain); PM2: Pathogenic Moderate (absent or extremely rare in population databases); PM5: Pathogenic Moderate (novel missense at same position as known pathogenic variant); PP2: Pathogenic Supporting (missense in gene with low rate of benign missense variation); PP3: Pathogenic Supporting (multiple computational tools predict damaging effect); PP5: Pathogenic Supporting (reported as pathogenic in reputable source); PP3-supp: PP3 applied at supporting strength level.

**Table 2:** Prevalence of dysmorphic facial features in this series.

Feature	This Series (*n* = 9)	HPO
Thin upper lip	9/9 (100%)	HP:0000219
Flat/smooth philtrum	8/9 (89%)	HP:0000319
Pointed chin	8/9 (89%)	HP:0000307
Short neck	8/9 (89%)	HP:0000470
Uniform long eyebrows	8/9 (89%)	HP:0004523
Wide nasal bridge	7/9 (78%)	HP:0000431
Long face	6/9 (67%)	HP:0000276
Broad forehead	6/9 (67%)	HP:0000337
Upslanting palpebral fissures	6/9 (67%)	HP:0000582
Bulbous nasal tip	6/9 (67%)	HP:0000414
Deep-set eyes	5/9 (56%)	HP:0000490
Long philtrum	5/9 (56%)	HP:0000343
Microcephaly	3/9 (33%)	HP:0000252
Other non-facial features		
Overweight (BMI ≥ 85th pct.)	5/9 (56%)	HP:0025502
Proximal placement of thumb	9/9 (100%)	HP:0009623

HPO: Human Phenotype Ontology; HP number: standardized phenotype identifier from Human Phenotype Ontology database (https://hpo.jax.org/ (accessed on 1 October 2025)).

**Table 3:** Summary of neurodevelopmental and clinical features.

Feature	P1	P2	P3	P4	P5	P6	P7	P8	P9 (M)
Sex	F	F	F	F	F	F	F	F	M
Global developmental delay	✔	✔	✔	✔	✔	✔	✔	✔	—
Hypotonia	✔	✔	✔	✔	✔	✔	✔	✔	—
Verbal communication	±	✔	—	—	✔	±	—	✔	✔
ASD features	✔	✔	✔	✔	✔	✔	✔	✔	—
Formal ASD (ADOS-2)	—	✔	—	✔	—	—	✔	—	—
Sleep disturbance	—	—	✔	✔	✔	—	✔	✔	±
Aggressive behavior	—	—	✔	✔	✔	—	✔	✔	—
Epilepsy	—	—	—	—	—	✔	—	—	—
Ventricular enlargement	✔	—	✔	✔	—	—	✔	✔	—
CC hypoplasia	—	—	—	✔	✔	—	✔	—	—
Intracranial cyst	—	✔	—	—	—	—	—	—	✔
Polymicrogyria	—	—	—	—	—	—	—	—	—

✔ present; — absent; ± mild presentation. F: female. M: male. CC: corpus callosum. ASD: autism spectrum disorder.

## Data Availability

The data presented in this study are available on request from the corresponding author.
